# Hemodynamic forces from 4D flow magnetic resonance imaging predict left ventricular remodeling following cardiac resynchronization therapy

**DOI:** 10.1186/s12968-023-00955-8

**Published:** 2023-08-25

**Authors:** Karin Pola, Anders Roijer, Rasmus Borgquist, Ellen Ostenfeld, Marcus Carlsson, Zoltan Bakos, Håkan Arheden, Per M. Arvidsson

**Affiliations:** 1 Clinical Physiology, Department of Clinical Sciences Lund, Lund University, Skåne University Hospital, Lund, Sweden; 2 Heart Failure and Valvular Heart Disease Section, Skåne University Hospital, Lund, Sweden; 3 Cardiology Division, Arrhythmia Section, Department of Clinical Sciences Lund, Lund University, Skåne University Hospital, Lund, Sweden

**Keywords:** Left bundle branch block, Device response, Heart failure with reduced ejection fraction, Pacemaker, Cardiac magnetic resonance

## Abstract

**Background:**

Patients with heart failure and left bundle branch block (LBBB) may receive cardiac resynchronization therapy (CRT), but current selection criteria are imprecise, and many patients have limited treatment response. Hemodynamic forces (HDF) have been suggested as a marker for CRT response. The aim of this study was therefore to investigate left ventricular (LV) HDF as a predictive marker for LV remodeling after CRT.

**Methods:**

Patients with heart failure, EF < 35% and LBBB (n = 22) underwent CMR with 4D flow prior to CRT. LV HDF were computed in three directions using the Navier–Stokes equations, reported in median N [interquartile range], and the ratio of transverse/longitudinal HDF was calculated for systole and diastole. Transthoracic echocardiography was performed before and 6 months after CRT. Patients with end-systolic volume reduction ≥ 15% were defined as responders.

**Results:**

Non-responders had smaller HDF than responders in the inferior-anterior direction in systole (0.06 [0.03] vs. 0.07 [0.03], p = 0.04), and in the apex-base direction in diastole (0.09 [0.02] vs. 0.1 [0.05], p = 0.047). Non-responders had larger diastolic HDF ratio compared to responders (0.89 vs. 0.67, p = 0.004). ROC analysis of diastolic HDF ratio for identifying CRT non-responders had AUC of 0.88 (p = 0.005) with sensitivity 57% and specificity 100% for ratio > 0.87. Intragroup comparison found higher HDF ratio in systole compared to diastole for responders (p = 0.003), but not for non-responders (p = 0.8).

**Conclusion:**

Hemodynamic force ratio is a potential marker for identifying patients with heart failure and LBBB who are unlikely to benefit from CRT. Larger-scale studies are required before implementation of HDF analysis into clinical practice.

**Graphical Abstract:**

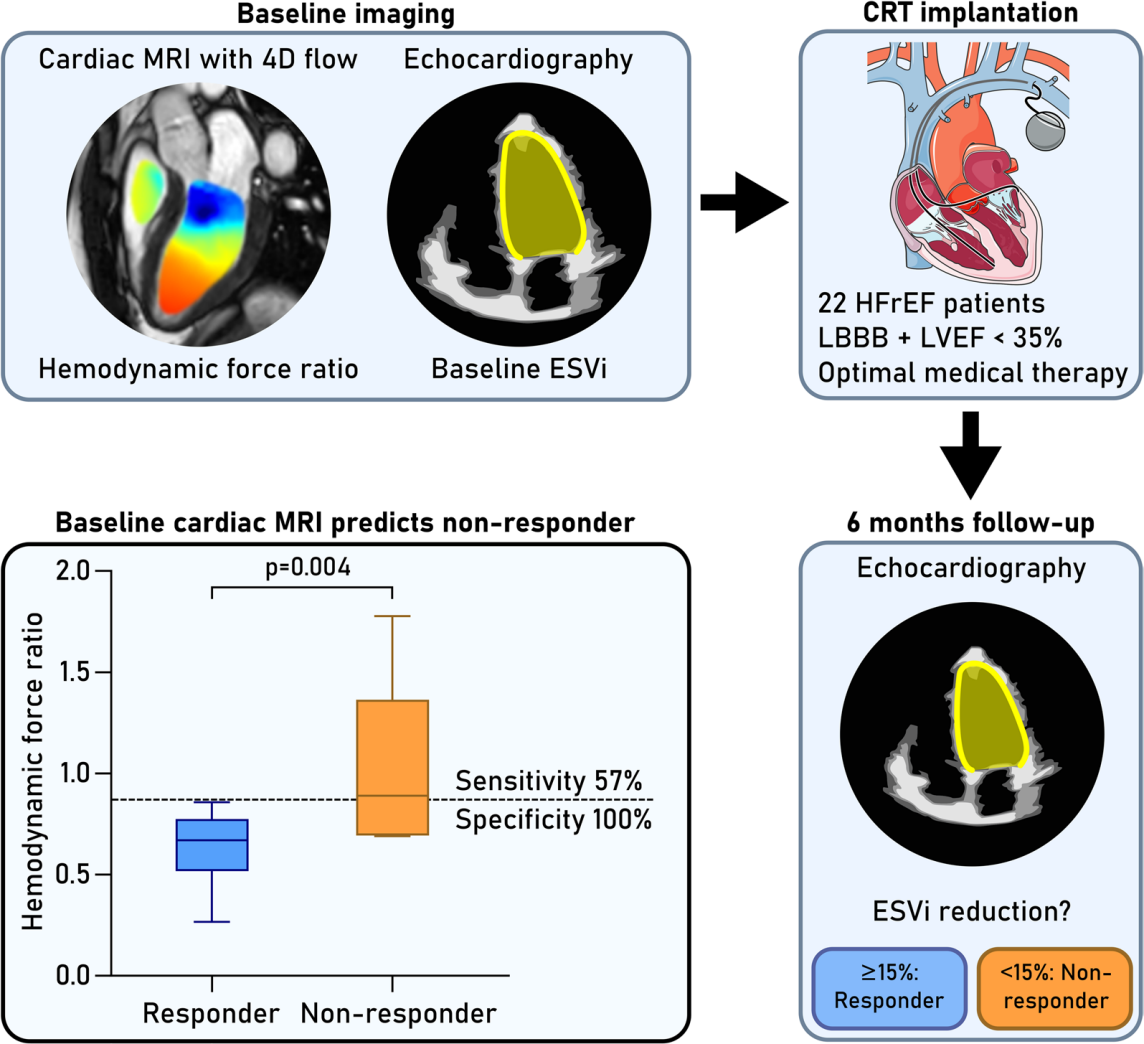

**Supplementary Information:**

The online version contains supplementary material available at 10.1186/s12968-023-00955-8.

## Background

Heart failure patients with left bundle branch block (LBBB) and ejection fraction (EF) < 35% despite optimal medical therapy may receive cardiac resynchronization therapy (CRT) to alleviate symptoms, improve quality of life, and reduce the risk of hospitalization and death [1–4]. Approximately one-third of patients show little or no benefit six months after CRT, despite attempts at improving patient selection and device implantation procedures [1, 2, 5–8]. The use of medical imaging to improve response rate has been repeatedly attempted with some success [9–12], however these analyses have not gained wide acceptance due to discouraging reproducibility and difficulty in implementing methods beyond the single-center setting [13, 14]. Better predictive markers for CRT response are therefore needed to reduce the number of unnecessary, expensive, and potentially harmful device implantations.

**Fig. 1:**
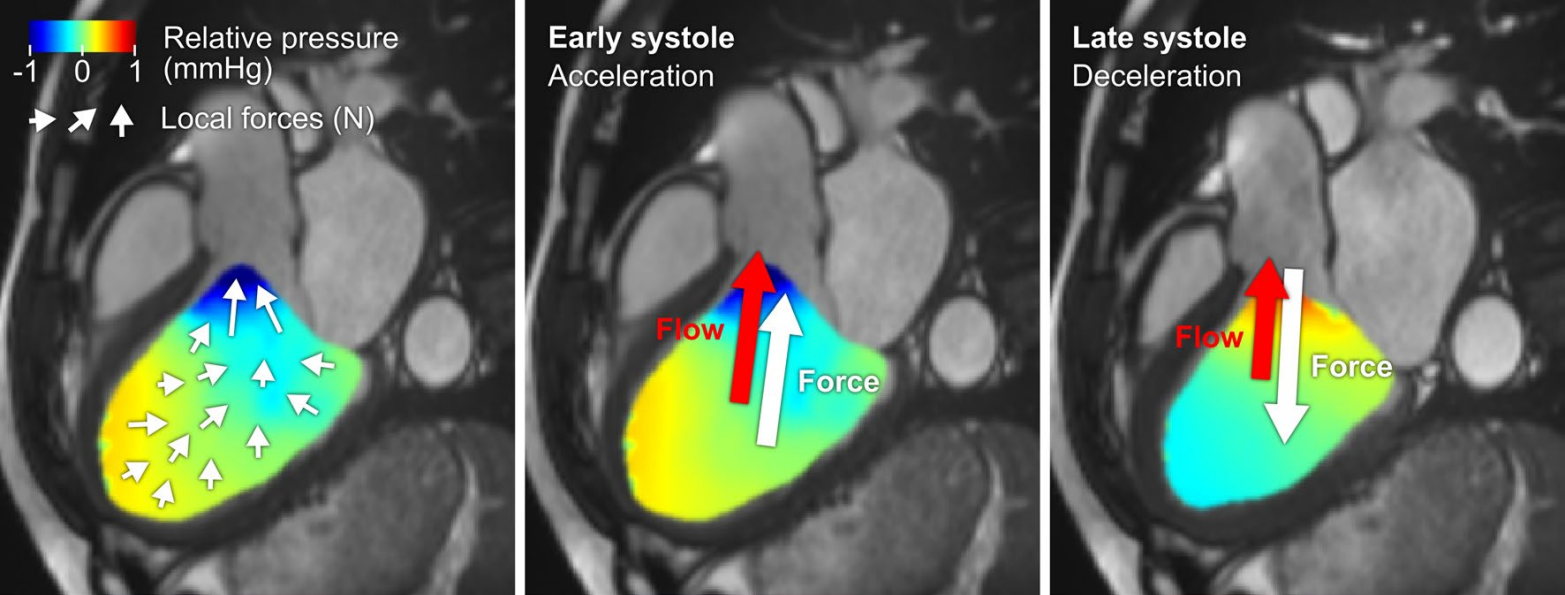
Pressure gradients, hemodynamic forces, and blood flow in a patient with heart failure and reduced ejection fraction. Left: The colored field illustrates the relative pressure gradients within the left ventricle at a single point in time. Local hemodynamic forces are illustrated with white arrows, with direction and magnitude indicated for each point. Center: The global force (white arrow) is the sum of all local forces and accelerates the blood flow (red arrow) towards the aorta during early systole. Right: By late systole, the global force is directed in the opposite direction of the flow, thereby decelerating the outflowing blood

Hemodynamic force (HDF) analysis of left ventricular blood flow is a novel marker of cardiac function suggested to convey unique information about the coupling between ventricular motion and the resulting blood flow patterns [15–18]. Hemodynamic forces constitute the net forces exchanged between the blood pool and surrounding myocardium, resulting from the sum of pressure gradients within the left ventricle (LV, Fig. 1). Several studies have established the feasibility and robustness of HDF measurements, showing a high accuracy and strong reproducibility for intraventricular HDF using three-dimensional, time-resolved (4D) flow cardiac magnetic resonance (CMR) imaging [17–20].

In healthy hearts, LV HDF are mainly oriented in the longitudinal direction, and increased ratio of transverse to longitudinal HDF indicate an abnormal blood flow pattern [16, 21–23]. In heart failure patients with LV dyssynchrony, HDF are largely independent of more traditional measures of ventricular function such as ECG QRS duration, EF, and strain measures, and HDF may thus provide added value for the individual assessment of patients eligible for CRT [22].

The aim of this study was therefore to investigate the value of hemodynamic force analysis as a predictive marker for CRT response in heart failure patients with LBBB, testing the hypothesis that HDF ratio before CRT implantation can identify patients who will not benefit from treatment.

## Methods

**Fig. 2:**
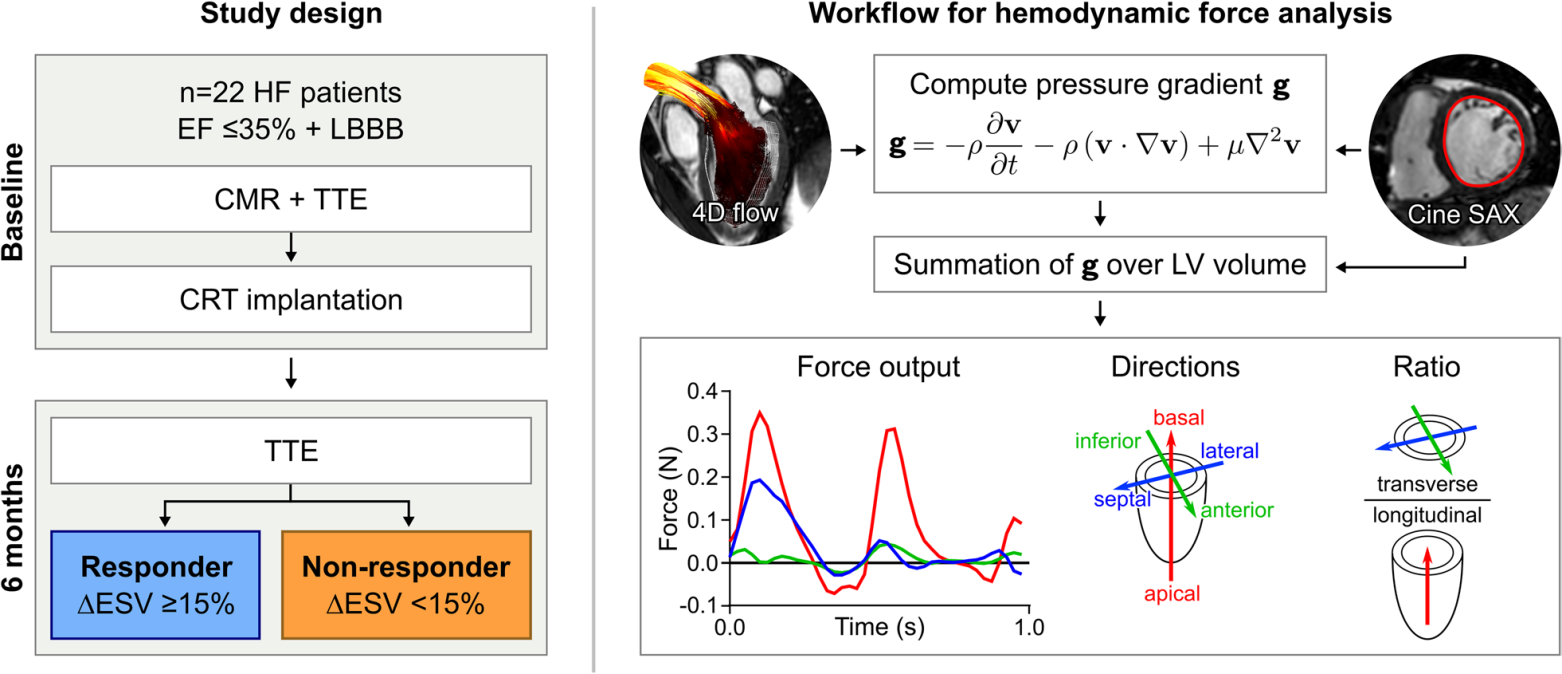
Study design and overview of workflow for hemodynamic force analysis. CMR, cardiovascular magnetic resonance; CRT, cardiac resynchronization therapy; ∆ESV, reduction in end-systolic volume; LBBB, left bundle branch block; TTE, transthoracic echocardiography

This study was a post-hoc analysis of patients with heart failure and LBBB from a previous prospective study evaluating outcome after cardiac resynchronization therapy (CRT Clinic; NCT01426321) [7]. Study design is summarized in Fig. 2. Inclusion criteria for the parent study were patients aged 18 and above with NYHA class II-IV heart failure with LBBB [24] and EF < 35% despite optimal medical treatment. Exclusion criteria were contradictions to CMR examination, atrial fibrillation, and > 10% aortic regurgitation.

Patients underwent transthoracic echocardiography (TTE) and CMR prior to CRT, and TTE six months post CRT. For HDF reference values, we also analyzed eight healthy subjects from our CMR research database, matched for sex and age at the group level. Left ventricular remodeling was assessed by echocardiography to determine patients’ response to CRT, where patients with a reduction of left ventricular end-systolic volume by ≥ 15% (using Simpson’s biplane method) at six months follow-up were classified as responders. All echocardiographic analysis was performed by one expert reader (AR).

The parent study sought to evaluate whether the use of medical imaging can improve CRT response, and 4D flow was an optional addition at the end of the CMR protocol. However, as the 4D flow acquisition at the time required approximately 30–40 min, and was not a crucial part of the main study, it was often not performed. For the present study, we therefore included all consecutive patients where 4D flow had been performed as part of the baseline CMR examination and where baseline and follow-up echocardiography data of adequate technical quality were available. A flowchart of the patient inclusion and exclusion from the parent to the present study is given in Additional file 1: Figure S1.

### CMR scan

Cardiovascular magnetic resonance images were acquired in patients at 1.5 T or 3 T (Achieva, Philips Healthcare, Best, The Netherlands) including ECG-gated short- and long-axis balanced steady-state free precession cine images, 4D flow, and late gadolinium enhancement (LGE) for viability. Healthy controls underwent CMR with a similar protocol except LGE at 1.5 T (Magnetom Aera, Siemens Healthcare, Erlangen, Germany). 4D flow was acquired from a volume covering the heart and proximal great vessels using a gradient echo sequence with Cartesian readout [25]. The 4D flow accuracy and precision across MRI vendors and using different sequence settings has been validated in vitro and in vivo [19, 26]. Typical scanner parameters: TE/TR 3.1–3.7 ms/5.1–6.3 ms, α 8°, VENC 100 cm/s, spatial resolution 3 mm isotropic, temporal resolution 50 ms, and acceleration factor 2 × 2 (phase × slice).

Viability was assessed by LGE imaging using a 3D phase sensitive inversion recovery sequence. Imaging was performed 10–20 min after intravenous administration of 0.2 mmol/kg gadolinium-based contrast agent (Dotarem, Guerbet, Roissy, France). Typical scanning parameters were: echo time 1.3 ms, effective repetition time 1/heartbeat, α 15°, spatial resolution 1.5 × 1.5 mm, slice thickness 8 mm. Inversion times were selected to provide optimal nulling of remote myocardium. LGE images were visually assessed for scar extent and transmurality using the AHA 17-segment model [7].

### CRT implantation procedure

All patients received a St. Jude Medical device (St. Paul, Minnesota, USA) with an atrial lead placed in the right atrial appendage and an RV lead placed in the apex or interventricular septum. Left ventricular electrode placement was targeted at the site of latest mechanical activation by echocardiography strain measurement, or at a suitable posterolateral mid or basal position at the discretion of the implanter. Coronary sinus cannulation was achieved with a steerable Medtronic Command sheath (Minneapolis, Minnesota, USA) or a pre-shaped access sheath together with guidewire and/or a diagnostic electrophysiology catheter, and sub-select sheaths were used if needed to place the lead in the desired branch.

### Data analysis

Image analysis was performed using the software Segment v3.3 R10057 (Medviso, Lund, Sweden) [27]. LV volumes were defined by semi-automated delineation of the endocardium in short-axis cine CMR images for the entire cardiac cycle [28].

Quality control of the 4D flow dataset included visual assessment of data quality in each of the three phase encoding directions as well as the magnitude images. Phase background errors were corrected using fitting to stationary tissue [29] and aliasing errors were corrected by phase unwrapping [30]. The spatial orientation of the cine images was adjusted to align with the 4D flow data. 4D flow was reconstructed to a through-plane flow stack in the same position as a 2D flow measurement in the ascending aorta to check for consistency in bulk flow.

### Hemodynamic forces

Hemodynamic forces were quantified using a validated method previously described in detail [18, 19]. Intraventricular pressure gradients from 4D-flow data were computed using the Navier–Stokes equations and integrated over the entire LV cavity (Fig. 2). Hemodynamic forces along three perpendicular axes were calculated from the field of pressure gradients, using a spatial reference system originating from the position of the atrioventricular (AV) plane. The apex-base direction was set as perpendicular to the AV plane, the lateral wall-septum direction was set as perpendicular to the apex-base direction and aligned to the LV outflow tract, and the inferior-anterior direction was set as perpendicular to both the apex-base and the lateral wall-septum directions. To facilitate visual comparison of HDF between subjects with different heart rates, a common time axis was created by linear resampling of the force curves for systole and diastole separately, where end systole was defined from linear extrapolation of the downward slope of the aortic flow curve, as previously described [31, 32].

### Statistical analysis

Root mean square (RMS) and peak forces were analyzed separately in the three directions, as in previous studies [18, 33]. Transverse to longitudinal HDF ratio was computed for systole and diastole as follows:

$$\text{Ratio} = \frac{\sqrt{{{\text{RMS}}^{2}_\text{lateral-septal}}{+ \text{RMS}^{2}_\text{inf-ant}}}}{{\text{RMS}}_\text{apex-base}}$$

Statistical analysis was performed using Prism v9.3.1 (GraphPad Software, La Jolla, California, USA). Continuous data is presented as median and interquartile range, and categorical data as absolute numbers and proportion (%). The Wilcoxon test was used for paired comparison of systolic and diastolic HDF ratio within the groups. The Mann–Whitney U test was used to compare unpaired continuous data between groups, and Fisher’s exact test was used to compare binary categorical data. Receiver operating characteristic (ROC) analysis and area under the curve (AUC) of HDF analysis was used to predict CRT response. A two-tailed p-value < 0.05 was considered significant.

## Results

### Patient characteristics

This study comprised 22 patients (17 men, 68 [7] years) examined at baseline with CMR and TTE between 2011 and 2014. At follow-up 6 ± 2 months post CRT, 15 patients were classified as responders and 7 as non-responders based on reduction of end-systolic volume ≥ 15% from TTE volumetry. Responders and non-responders had similar baseline LV volumes and mass, cardiac output, cardiac index, and heart rate, and similar proportion of women, QRS duration > 150 ms, and body mass index > 30 kg/m^2^ (Table 1). A trend was seen towards ischemic heart disease being more frequent in non-responders than responders (86% vs. 40%), although the difference was not statistically significant (p = 0.07). Heart-failure related hospitalization and mortality within 5 years did not differ between responders and non-responders (Table 1).

**Table 1:** Patient characteristics and cardiac volumes measured from CMR

	Responders (n = 15)	Non-responders (n = 7)	p-value Responders vs. Non-responders	Controls (n = 8)	p-value all patients vs. controls
Age, years	67 [8]	70 [7]	0.822	65 [2]	0.103
Male, n (%)	11 (73)	5 (71)	1.0	4 (50%)	0.384
BMI > 30 kg/m^2^, n (%)	1 (7)	2 (29)	0.227	0	–
QRS > 150 ms, n (%)	14 (93)	5 (71)	0.227	0	–
IHD etiology, n (%)	6 (40)	6 (86)	0.074	0	–
DCM etiology, n (%)	9 (60)	1 (14)	0.074	0	–
Previous myocardial infarction, n (%)	4 (27)	5 (71)	0.074	0	–
Heart-failure related hospitalization within 5 years, n (%)	2 (13)	0	1	0	–
Mortality within 5 years, n (%)	1 (7)	1 (14)	1	0	–
Blood pressure systole/diastole, mmHg	137 [34]/78 [17]	120 [15]/73 [5]	0.321/0.206	127 [10]/77 [3]	0.608/0.435
Diabetes, n (%)	1 (7)	1 (14)	1.0	0	–
Beta blocker, n (%)	14 (93)	6 (86)	1.0	0	–
ACEi or ARB, n (%)	15 (100)	7 (100)	1.0	0	–
Platelet inhibitor, n (%)	4 (27)	4 (57)	0.343	0	–
Diuretics, n (%)	6 (40)	4 (57)	0.652	0	–
Lipid-lowering drug, n (%)	6 (40)	6 (86)	0.074	0	–
Heart rate, bpm	66 [14]	62 [11]	0.179	65 [16]	0.774
LV EDV, ml	286 [130]	348 [78]	0.448	158 [31]	–
LV EDVi, ml/m^2^	156 [80]	156 [39]	0.731	87 [6]	–
LV ESV, ml	197 [87]	238 [77]	0.783	68 [11]	–
LV ESVi, ml/m^2^	106 [50]	115 [45]	0.891	36 [5]	–
LV SV, ml	77 [24]	96 [30]	0.162	91 [24]	0.420
LV SVi, ml/m^2^	39 [15]	47 [12]	0.407	49 [6]	0.170
LV EF, %	28 [9]	31 [9]	0.630	57 [2]	–
CO, l/min	5.1 [1.5]	5.1 [1.9]	0.837	5.3 [1.0]	0.696
CI, l/min/m^2^	2.7 [0.75]	2.6 [0.73]	1.0	2.9 [0.56]	0.185
LGE positive, n (%)	11 (73)	6 (86)	1.0	–	–
LGE mean % extent/transmurality	31/42	44/52	0.334/0.360	–	–
LGE positive in septal segments (2, 3, 8, 9 or 14), n (%)	5 (33)	4 (57)	0.376	–	–

Data is expressed as median and interquartile range [IQR] or absolute values and percentage (%). Data for medications show the usage at 6-months follow-up. *ACEi* angiotensin converting enzyme inhibitor, *ARB* angiotensin II receptor blocker, *BMI* body mass index, *CI* cardiac index, *CO* cardiac output, *DCM* dilated cardiomyopathy, *IHD* ischemic heart disease, *bpm* beats per minute, *EF* ejection fraction, *LV EDVi* left ventricular end-diastolic volume indexed to body surface area, *ESV* end-systolic volume, *LGE* late gadolinium enhancement, *LVM* left ventricular mass, *SV* stroke volume

### Hemodynamic forces

**Fig. 3:**
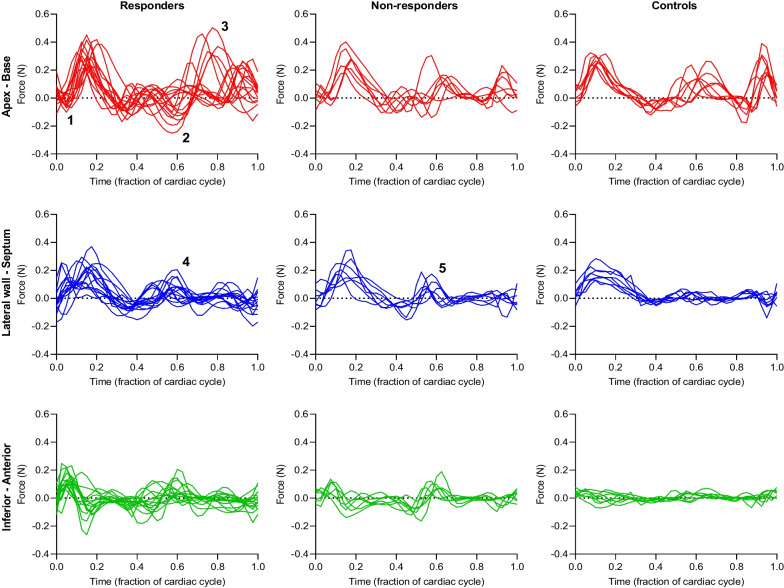
Left ventricular hemodynamic forces over single cardiac cycle. In the apex-base direction, responders typically had a negative impulse at the beginning of systole (1), which is not seen as prominently in non-responders or controls. A subgroup of responders (n = 6) had a pattern with larger force amplitudes in diastole, with an early negative impulse and a late positive impulse (2 and 3). In the lateral wall-septum direction responders and non-responders had force patterns with a larger positive impulse in early diastole compared to controls (4 and 5). In the inferior-anterior direction, responders typically had larger amplitudes throughout the cardiac cycle compared to non-responders and controls

Left ventricular force patterns over one cardiac cycle for responders, non-responders and controls are presented in three orthogonal directions in Fig. 3. Data for controls is presented as a reference and compared to all patients combined in one group. Differences between responders and non-responders were found in all three directions.

**Fig. 4:**
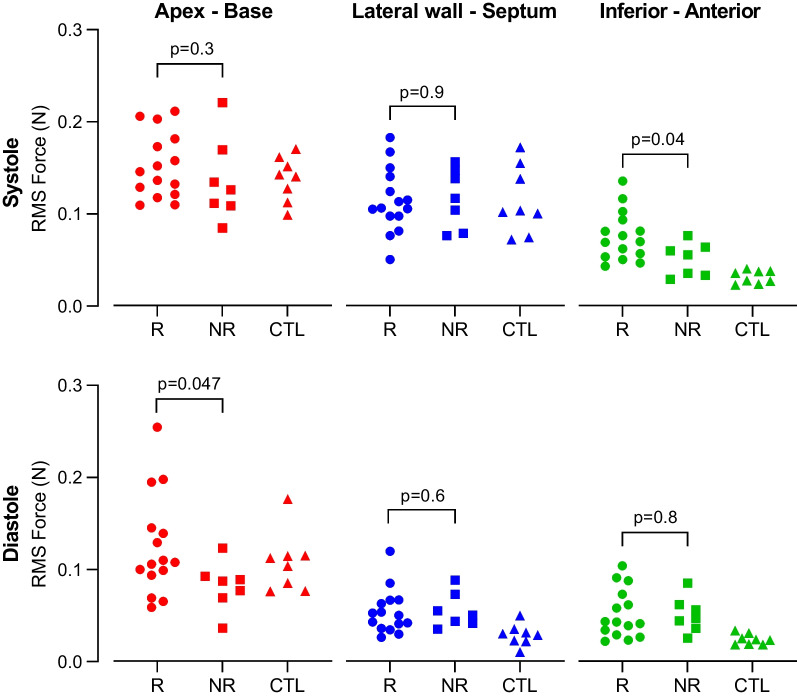
Root mean square (RMS) left ventricular hemodynamic forces in responders (R, circles) and non-responders (NR, squares) to cardiac resynchronization therapy, and controls (CTL, triangles). Responders had significantly higher systolic HDF in the inferior-anterior direction, and significantly higher diastolic longitudinal (apex-base) HDF compared to non-responders

Root mean square (RMS) HDF are presented in Fig. 4.

Systolic HDF in the inferior-anterior direction were smaller in non-responders compared to responders (RMS: 0.055 [0.027] vs. 0.070 [0.032], p = 0.039; peak: 0.11 [0.057] vs. 0.15 [0.079], p = 0.032), but no difference between the groups was found in any other direction (apex-base RMS: 0.13 [0.042] vs. 0.15 [0.052], p = 0.298; peak: 0.27 [0.10] vs. 0.31 [0.14], p = 0.185) (lateral wall-septum RMS: 0.12 [0.051] vs. 0.11 [0.035], p = 0.891; peak: 0.21 [0.10] vs. 0.22 [0.10], p = 0.837). Controls had smaller systolic HDF compared to the group of all patients in the inferior-anterior direction (RMS: 0.031 [0.013] vs. 0.063 [0.032], p < 0.0001; peak: 0.061 [0.017] vs. 0.13 [0.069], p < 0.0001), but no difference between the groups was found in any other direction (apex-base RMS: 0.14 [0.043] vs. 0.14 [0.059], p = 0.801; peak: 0.29 [0.051] vs. 0.30 [0.16], p = 0.662) (lateral wall-septum RMS: 0.10 [0.070] vs. 0.11 [0.049], p = 0.662; peak: 0.17 [0.073] vs. 0.22 [0.12], p = 0.185).

Diastolic HDF in the apex-base direction were smaller in non-responders compared to responders for RMS (0.087 [0.018] vs. 0.11 [0.046], p = 0.047), but did not differ for peak (0.16 [0.054] vs. 0.26 [0.10], p = 0.091). No difference in diastolic HDF was found in any other direction for either RMS or peak (lateral wall-septum RMS: 0.050 [0.022] vs. 0.050 [0.026], p = 0.630; peak: 0.13 [0.068] vs. 0.10 [0.050], p = 0.448) (inferior-anterior RMS: 0.047 [0.019] vs. 0.043 [0.036], p = 0.783; peak: 0.10 [0.040] vs. 0.10 [0.069], p = 0.582). Controls had smaller diastolic HDF compared to the group of all patients in the lateral wall-septum direction (RMS: 0.029 [0.012] vs. 0.050 [0.027], p = 0.0004; peak: 0.057 [0.019] vs. 0.11 [0.066], p = 0.0009), and in the inferior-anterior direction (RMS: 0.023 [0.011] vs. 0.043 [0.032], p = 0.0002; peak: 0.054 [0.024] vs. 0.10 [0.062], p = 0.0009), but HDF did not differ between the groups in the apex-base direction (RMS: 0.11 [0.036] vs. 0.099 [0.057], p = 0.872; peak: 0.22 [0.15] vs. 0.24 [0.16], p = 0.872).

**Fig. 5:**
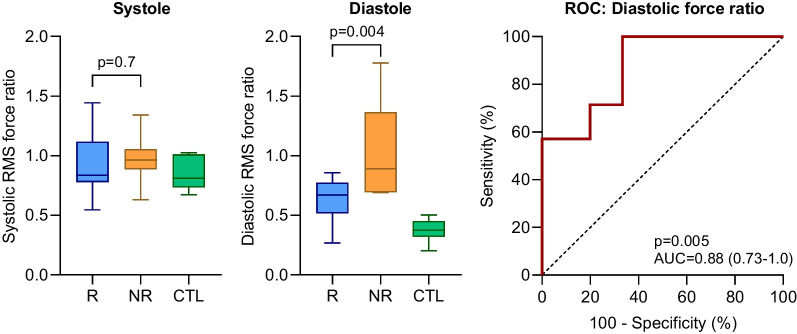
Ratio of transversal to longitudinal hemodynamic forces during systole (left) and diastole (center) in responders (R, blue) and non-responders (NR, orange) to cardiac resynchronization therapy, and controls (CTL, green). Whiskers show range. Right: Receiver operating characteristic curve for ratio of transversal to longitudinal root mean square (RMS) forces in diastole. Dashed line of equality

The ratio of transversal to longitudinal RMS HDF is presented in Fig. 5. In systole, no difference was found between non-responders and responders (RMS: 0.96 [0.11] vs. 0.84 [0.28], p = 0.731; peak: 0.95 [0.24] vs. 0.81 [0.44], p = 0.731). No difference in systolic ratio was found between controls and the group of all patients for RMS (0.81 [0.28] vs. 0.95 [0.27], p = 0.368), but controls had smaller peak ratio compared to the group of all patients (0.63 [0.19] vs. 0.83 [0.41], p = 0.040). In diastole, non-responders had larger ratio compared to responders (RMS: 0.89 [0.45] vs. 0.67 [0.20], p = 0.004; peak: 1.1 [0.36] vs. 0.57 [0.33], p = 0.011). Controls had smaller diastolic ratio compared to the group of all patients (RMS: 0.37 [0.13] vs. 0.69 [0.23], p = 0.0002; peak: 0.39 [0.17] vs. 0.66 [0.53], p = 0.003). Spearman correlation analysis showed that diastolic force ratio in patients did not correlate with QRS width (p = 0.196), stroke volume (p = 0.304), ejection fraction (p = 0.462), cardiac output (p = 0.856), or age (p = 0.810).

Receiver operating characteristic (ROC) analysis of diastolic RMS HDF ratio found an area under the curve (AUC) of 0.88 (p = 0.005). Identification of non-responders using diastolic RMS HDF ratio with a specificity of 100%, resulted in a ratio of > 0.87 for non-responders, with a sensitivity of 57% (Fig. 5, right panel). For diastolic peak ratio, AUC was 0.84 (p = 0.012).

**Fig. 6:**
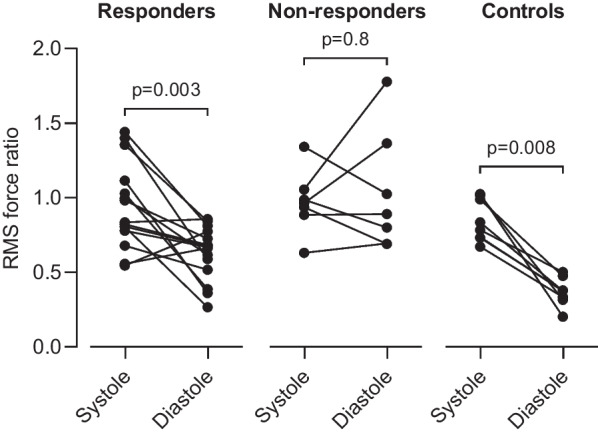
Paired comparison of transversal to longitudinal root mean square hemodynamic force ratio in responders (left) and non-responders (middle) to cardiac resynchronization therapy, and in controls (right)

Intragroup comparison of RMS HDF ratio between systole and diastole found no difference in non-responders (p = 0.813, Fig. 6), in contrast to responders and controls where the ratio was larger in systole than in diastole (p = 0.003 and p = 0.008 respectively). For peak HDF ratio, there was no statistically significant difference between systole and diastole in responders (p = 0.055) or non-responders (p = 0.469), but controls had larger ratio in systole than in diastole (p = 0.002).

## Discussion

In this prospective, post hoc study on heart failure patients eligible for cardiac resynchronization therapy, we found that the ratio between transversal and longitudinal hemodynamic forces in diastole could identify non-responders with a sensitivity of 57% and a specificity of 100%. Our data imply that diastolic blood flow patterns may be of additional value to standard systolic parameters in assessing LV function in LBBB. Hemodynamic force analysis from 4D flow CMR may therefore improve detection of patients less likely to exhibit reverse remodeling after CRT despite fulfilling current selection criteria.

### Systole

In systole, responders had larger HDF compared to non-responders in the transverse inferior-anterior direction. This emphasizes the value of 4D flow CMR over other methods for HDF measurements which are less sensitive to aberrant forces in this direction [20, 34]. The healthy heart typically has large systolic HDF amplitudes in the apex-base and lateral wall-septum directions, and small HDF in the inferior-anterior direction for optimal pumping efficiency. Large forces in the inferior-anterior direction can thus be interpreted as an indicator of an inefficient pumping mechanism, as they reflect systolic blood flow not optimally aligned with the LV outflow tract. As CRT aims to increase cardiac output by improving LV contraction synchrony, larger transverse forces in responders could indicate a pathophysiology that will benefit from CRT. The similarity in systolic HDF between responders and non-responders in the apex-base and inferior-anterior directions could be expected since all patients met the current CRT selection criteria, which focus on systolic function.

### Diastole

In diastole, the patient group displayed pronounced HDF aberrations, in line with previous results [22, 23]. Interestingly, for diastolic force ratio our data indicate that responders may have less deranged HDF compared to non-responders, and in fact bear some resemblance to healthy hearts, while non-responders may be identified by increased diastolic HDF. This somewhat unexpected finding is further strengthened by comparison of the force ratios in systole and diastole, where responders and controls had larger force ratio in systole, while non-responders had similar force ratios in systole and diastole. We speculate that while LBBB leads to systolic dysfunction through disorganized ventricular depolarization, differences in diastolic HDF between responders and non-responders indicate a varying impact on ventricular relaxation, which leads to altered intraventricular hemodynamics and further worsens the ability to maintain normal cardiac output, perfusion, and myocardial tissue energetics. By extension, CRT may therefore be best suited to treat patients whose systolic dysfunction has less pronounced coupling to relaxation abnormalities, as represented by seemingly normal diastolic HDF in responders but deranged in non-responders. In this patient cohort, computation of HDF could thus be a sensitive marker of aberrant diastolic hemodynamics.

### ‘Responder’ vs. ‘non-responder’

Hemodynamic force analysis may identify patients unlikely to respond with reverse volumetric remodeling following CRT. Some of these patients would possibly benefit from more aggressive medical treatment, while others are likely to deteriorate further without CRT, complicating the term ‘non-responder’. While CRT is considered a cost-effective treatment, the beneficial effects manifest well beyond volumetric response [14], as improved exercise capacity and quality of life, reduced HF hospitalization and ultimately, reduced mortality [3]. Establishing a rule-out criterion for treatment is therefore more complex than determining the expected end-systolic volume change, which is only a proxy for improved myocardial energetics and subsequent outcomes. Our choice to use echocardiographic end-systolic volume reduction as the outcome measure was primarily motivated by the availability of previously acquired data. As a total of only two all-cause deaths were noted in the patient cohorts, our study was insufficiently powered to evaluate hard outcomes. Within these limitations, we therefore submit that diastolic HDF ratio computed from 4D flow CMR is a potential marker complementary to current clinical selection criteria for identifying CRT volumetric non-response. We consider this a proof-of-concept study of limited scope whose results cannot be immediately transferred to either long-term hard outcomes or clinical response. Whether HDF analysis offers sufficient granularity to predict clinical and hard outcomes after CRT remains to be evaluated in a larger study.

### Relation to established clinical predictors of CRT outcome

Previous studies have suggested female sex, BMI < 30, QRS-duration > 150 ms, septal LGE, and no prior myocardial infarction as predictors associated with improved outcome of CRT at the group level [35, 36]. In our small study, there were no statistically significant differences between responders and non-responders for these parameters, which strengthens the hypothesis of HDF as a potentially more powerful marker in this patient cohort. Previous work has also found HDF ratio to be independent of established markers of LV dyssynchrony [22].

### Limitations

This study included a small patient cohort and few female subjects, and HDF analysis as a prognostic marker for CRT response requires further validation in larger-scale studies before clinical implementation. Ischemic etiology of heart failure is a potential confounder as it was more frequently observed in non-responders than responders, which stresses the need for reproduction of the study with a larger patient cohort. Despite the small number of subjects, distinct differences in HDF patterns were found between responders, non-responders, and controls, in line with previous results suggesting improved HDF alignment in patients with volumetric response at 4–11 months follow-up [21].

Acquisition of HDF from 4D flow CMR within clinically feasible timescales is achieved through relatively low acquired spatial and temporal resolutions [37]. Phantom and in vivo validation found 4D flow CMR typically underestimates both RMS and peak HDF by about 15% compared to the in vitro reference standard, laser particle imaging velocimetry with high spatiotemporal resolution [19]. While clinical implementation of 4D flow CMR has historically been impeded by relatively long acquisition times, contemporary accelerated sequences offer whole-heart acquisition times of 5–10 min with preserved data quality [19, 38], and 4D flow CMR remains the gold standard for HDF analysis [19, 20, 31].

It would have been desirable to measure HDF at follow-up, to evaluate whether force realignment correlates with treatment effect, as recently suggested from echocardiographic studies [15, 21, 39]. The presence of intracavitary pacemaker leads will likely impair 4D flow data integrity, as this technique is sensitive to field inhomogeneities. Under such conditions HDF may be estimated from endocardial boundary dynamics [40] from gradient recalled echo images instead. While this approach is less precise than HDF computed from 4D flow [20], it may be applied in the presence of magnetic field inhomogeneities as long as the endocardial boundary can be clearly distinguished, and therefore remains a viable option for future studies post CRT.

## Conclusion

Hemodynamic force ratio is a potential marker for identifying heart failure patients with left bundle branch block who are unlikely to benefit from cardiac resynchronization therapy. Larger-scale studies are required before implementation of hemodynamic force analysis into clinical practice.

## Supplementary Information

**Additional file 1: Figure S1**. Inclusion and exclusion flowchart.

## Data Availability

Processed data underlying this article may be shared on reasonable request to the corresponding author.

## Abbreviations

4D flow: Three-dimensional, three-component, time-resolved flow
AUC: Area under the curve
AV plane: Atrioventricular plane
CRT: Cardiac resynchronization therapy
HDF: Hemodynamic force
LBBB: Left bundle branch block
RMS: Root mean square
ROC: Receiver operator characteristic
TTE: Transthoracic echocardiography
