# Supplementary figures and images for: Hemodynamic forces from 4D flow magnetic resonance imaging predict left ventricular remodeling following cardiac resynchronization therapy
Source: J Cardiovasc Magn Reson. 2023 Aug 25;25:45. doi: 10.1186/s12968-023-00955-8 (PMC10463519; doi:10.1186/s12968-023-00955-8)

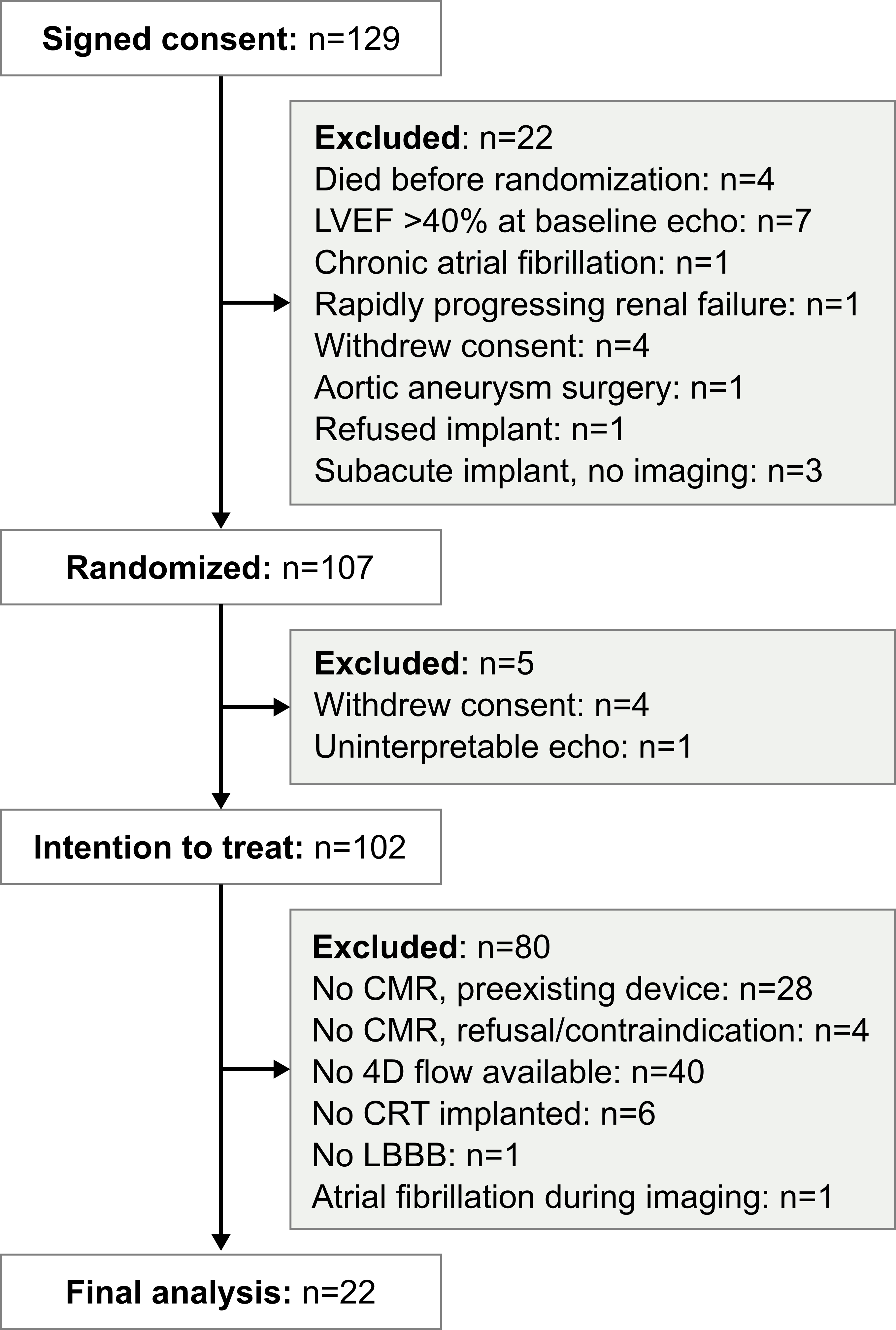

Supplement: Supplementary file 1 — Additional file 1: Figure S1. Inclusion and exclusion flowchart. [file 12968_2023_955_MOESM1_ESM.png]
